# Application and performance of a Low Power Wide Area Sensor Network for distributed remote hydrological measurements

**DOI:** 10.1038/s41598-023-45474-9

**Published:** 2023-10-23

**Authors:** Scott J. Ketcheson, Vitaly Golubev, David Illing, Bruce Chambers, Sheldon Foisy

**Affiliations:** 1 Faculty of Science and Technology, Athabasca University, Athabasca, Canada; 2 Athabasca River Basin Research Institute, Alberta, Canada; 3 Riot Technology Corp., North Saanich, Canada; 4 BICS, Brussels, Belgium

**Keywords:** Environmental sciences, Hydrology, Characterization and analytical techniques

## Abstract

Communication distances of wireless sensor networks (WSNs) are greatly limited in settings where vegetation coverage is moderate or dense, and power consumption can be an issue in remote environmental settings. A newer innovative technology called “Low Power Wide Area Sensor Networks” (LPWAN) is capable of greater communication distances while consuming less power than traditional WSNs. This research evaluates the design and in-field performance of a LPWAN configuration in headwater catchments to measure environmental variables. The performance of the Beta LPWAN deployment indicate reduced signal strength in topographic valleys, but better actual than modelled data transmission performance. System performance during extreme cold temperatures (below – 15 ºC) resulted in increased sensor down time. The configuration of antennae combinations provides the greatest improvement in signal strength and system performance. This technology facilitates remote collection of physically-based, spatially-distributed information within regions with limited accessibility, ultimately advancing data collection capabilities into areas that are not feasible to visit regularly.

## Introduction

Wireless sensor networks (WSN) developed over the preceding decade have demonstrated capabilities to provide a cost-effective and reliable means to collect spatially representative (distributed) hydrological data in near real time^1–3^. WSNs are smart wireless mesh networks composed of distributed instrument clusters (referred to as sensor nodes or “motes”) and a primary “mesh network manager”, or “gateway”, that automatically coordinates the wireless network of individual sensor nodes. However, communication distances between adjacent sensor nodes and the network manager becomes greatly limited in settings where vegetation coverage is moderate or dense^1,2^. Additionally, power consumption of some WSN systems is relatively high^1^ and can be an issue for deployment in settings relying on portable power supplies. This reduces the geographical area that can be effectively covered by the sensor network, hence limiting the spatial distribution of the instrumentation, consequently reducing the applicability of deploying WSNs for environmental research and/or monitoring campaigns.

In part driven by the shortfalls of WSN technology, an innovative new technology has since been developed (by Riot Technology; RT) that is capable of much greater communication distances between individual sensor nodes, while consuming far less power than traditional WSN technology. This innovative new technology, called “Low Power Wide Area Sensor Networks” (LPWAN), can facilitate more practical deployments of instrument clusters across larger spatial areas and with less logistical constraints for power supply than the older WSN technology. Out of necessity, the development, optimization and initial range assessments and field-testing of the communication technology itself was completed in the lab and then in urban settings out of convenience. This research represents one of the first field-deployments of a Beta version of the innovative LPWAN technologies in a remote, research-based environmental setting. In addition to the typical performance variances and trade-offs between radio parameters and antennae configurations, this research represents an actual real-world field-deployment where vegetation, weather and topography will impact communication capabilities and power consumption. It is critical to understand the actual performance of the LPWAN technologies in a field setting in order to assess the suitability for remote research and/or monitoring applications, and to improve and optimize future designs and radio parameter configurations. Environmental scientists and researchers across academia, government and private industry are potential end-users of this technology. Accordingly, it is imperative to understand the conditions under which these systems excel, and potential limitations should be known. Therefore, the goal of the research is to inform and improve appropriate application and deployment of LPWAN technology by end-users in the future, ensuring that the full capabilities of the systems are utilized.

### Contextual challenge

Advancing knowledge in the hydrological sciences often requires accessing remote regions to facilitate instrument installation and measurement of numerous hydrological variables to understand the dominant governing controls and processes that dictate how a particular ecosystem functions. However, accessing and instrumenting areas of potential hydrological importance can be both expensive and logistically challenging, and there has been a shift in hydrology-related publications away from field-based studies and towards computer-based simulations and modelling^4^. Accordingly, there is a critical lack of information within potentially important but relatively inaccessible areas of the landscape. Thus, although hydrological modelling and analyses of large-scale streamflow data can highlight the regional importance of water generated within these inaccessible areas^5,6^, there remains a poor understanding of the hydrological processes operating locally^7,8^. Enhanced data collection enabled by WSN technology can reduce the number of model parameters required by 50% when applied in conjunction with low complexity hydrological modelling^9^. For example, WSN technology can enable prediction of streamflow and nutrient export from agriculturally-dominated catchments^9^. Hence, WSN technology allows the application of streamflow prediction models to be more proactive rather than reactive^10^. However, communication distances between individual sensor nodes are constrained, with a conservative distance of up to 50 m in challenging environmental settings^2^. Hence, application across broad areas to capture local hydrological processes throughout even small catchments (e.g., < 5 km^2^) becomes unrealistic or cost prohibitive. These local processes are key components to understanding ecosystem functioning and identifying which components of these ecosystems are particularly sensitive to human or climate-induced change. Consequences of this knowledge deficit are reflected in hydrological modelling capabilities, where modelling performance can be poor in regions with little physically-based information to adequately parameterize the regional-scale modelling^6^. For example, regional model performance in Canada’s Athabasca River Basin is weak in the mid-to-lower reaches of the basin^11,12^, owing in part to a critical lack of understanding of the nature of the water generation mechanisms that contribute to the observed regional-scale downstream flow patterns. Thus, since inaccessibility does not dictate hydrological importance, it is imperative that a solution is developed that facilitates the measurement of environmental variables in remote settings in order to help build an understanding of the function and importance of ecosystems that are relatively difficult to access. Therefore, the objective of this research was to understand how much different factors (e.g., topography, weather, land cover) affect the performance of LPWAN systems, and to provide information and recommendations to inform and optimize future designs.

### Low power wide area sensor networks: the solution?

Key LPWAN Advantages:

- requires only one datalogger setup to service multiple instrumentation/measurement locations
  o data transmission and cellular usage cost-savings
- improved communication distances
  o up to nearly 2 km in the current study
- reduced power consumption
- implementation of a wireless bridge for SDI-12 communications
- facilitates near real-time remote collection of spatially distributed datasets in areas with limited accessibility

Effective environmental monitoring requires regular measurements of environmental variables under different conditions (e.g., wet vs. dry) and seasons (e.g., summer vs. winter). However, this is not always possible in locations that are remote and/or difficult to access^13–15^. The older WSN technology communication distances are limited, especially in vegetated areas, and power consumption can be relatively high^1,2^. Additionally, there can be unpredictable differences between the expected communication range under ideal conditions and the actual performance and true range realized in application in the field. For example, a WSN-based buoy network platform used WSN technology with a 3–4 km maximum transmission range under ideal conditions^16^; however, the realized range once the system was deployed in the field was approximately 400 m^17^. More traditional instrumentation, measurement techniques and experimental designs are constrained by logistical challenges (e.g., cable length, power requirements, datalogger costs). While WSNs provide a small-scale solution to these issues, the LPWAN systems tested in this study address the remaining issues within WSNs by improving communication distances and reducing power consumption. This new technology is based on an Internet of Things (IoT) long-range wireless infrastructure using LoRa® (short for LongRange) Technology. LoRa prioritizes transmission distance and low power consumption over data volume, so it is well suited to sensor networks with small data packets. It operates in the 900 MHz Industry Scientific and Medical (ISM) band (902–928 in North America) and utilizes Chirp Spread Spectrum (CSS). It operates best in line-of-sight conditions (i.e., no physical obstructions between transmitter and receiver), with signal losses expected with increased obstructions occupying the line-of-sight communication pathway.

More traditional, typical stand-alone datalogger systems each require an external power source and charging capabilities (e.g., 12 V battery with solar panel), weather-proof enclosure and independent telemetry capabilities, if desired. However, a field-deployment of a LPWAN system requires only one datalogger setup to service multiple instrumentation/measurement locations spatially distributed over kilometers away from the central datalogger system. One of the key aspects of the RT LPWAN system is the implementation of a wireless bridge for SDI-12 communications. The SDI-12 protocol requires very stringent response times that wireless systems are typically unable to meet due to the wireless transmission latency. Novel techniques are employed in the RT radios to transparently account for the wireless latency issues. Additionally, the central datalogger setup can be equipped with telemetry (e.g., cellular modem or satellite communications), which provides near real-time remote data collection capabilities from multiple measurement locations throughout a study site via one single external telemetry setup. This provides considerable data transmission and cellular usage cost-savings as compared to equipping all measurement locations with a traditional datalogger and individual telemetry capabilities and minimizes the need for stand-alone datalogger systems.

The innovative methodological approach here combines field performance with environmental conditions and physical features along communication lines, which allows us to identify and begin to characterize features that influence and control operational capabilities of the LPWAN systems. The main challenge that this research addresses is to understand the underlying controls on the communications capabilities of the LPWAN technology in a field-based setting. Challenges in advancing from the technology development stage to field-deployment include being able to adapt to topographical, vegetation and environmental variables a priori, for this technology to be applicable to environmental researchers and practitioners in the future. Thus, this research includes an evaluation of communication performances as related to detailed landscape (e.g., distance, topography and vegetation) and meteorological (e.g., rain events, air temperature) conditions to provide a comprehensive evaluation of the underlying variables that govern communication capabilities of the LPWAN technology.

## Materials and methods

### Study site

**Figure 1:**
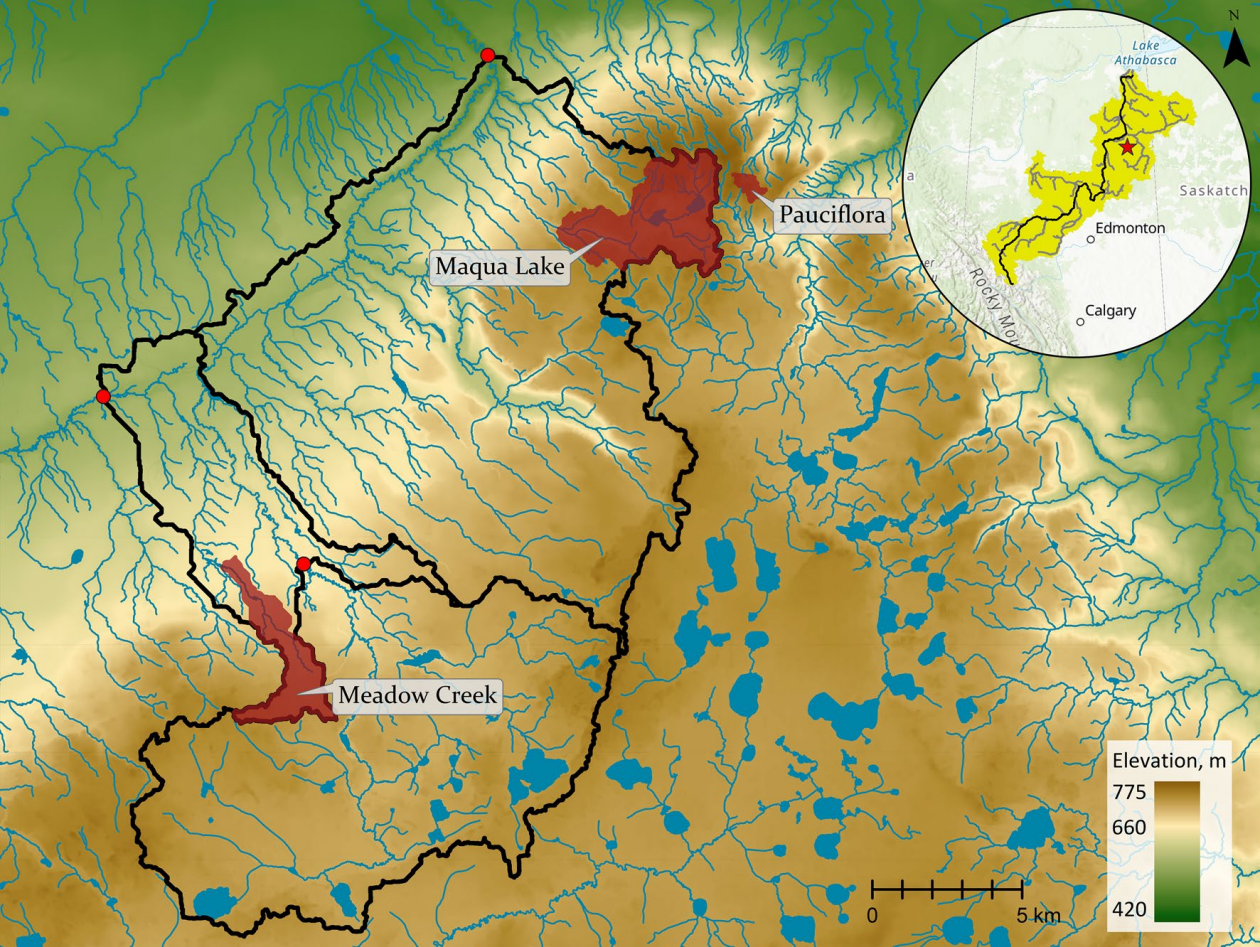
The Stony Mountain Headwater Catchment Observatory (map generated using ESRI ArcGIS Pro 2.2; www.esri.com).

The Stony Mountain Headwater Catchment Observatory (SMHCO; Fig. 1) comprises five small (< 10 km^2^) wetland-dominated headwater catchments located on the Stony Mountain Boreal upland landform ~ 40 km south-east of Fort McMurray, Alberta, Canada. The Stony Mountain Uplands span approximately 600–850 m above sea level (masl), with the SMHCO catchments varying in elevation from 653 to 767 masl^18^. Individual catchment terrain includes topographic variability from 24 to 65 m change in elevation. Each catchment contains a central peatland ecosystem situated in the topographic low or central valley, bordered by adjacent forested upland ecosystems comprising diverse mixedwood forests. Peatlands within the SMHCO include two moderate-rich basin fens (ground cover dominated by *Sphagnum* spp.; tree cover predominantly *Larix laricina*), a poor basin fen (predominantly *Sphagnum* spp. and ericaceae shrubs), a channel fen (dominated by graminoids) and a thicket swamp-peatland (graminoids with a dense cover of shrubs and small trees dominated by *Salix*, *Alnus* and *Betula* spp.). Forests on moist low-gradient slopes proximal to the adjacent low-lying peatland are typically densely treed and dominated by *Picea mariana*. These densely treed regions often transition to a more sparse mixedwood deciduous tree cover dominated by *Picea banksiana* and *Populus tremuloides* at higher elevations, with tree heights from ~ 10 to 25 m.

### LPWAN deployment at SMHCO

**Figure 2:**
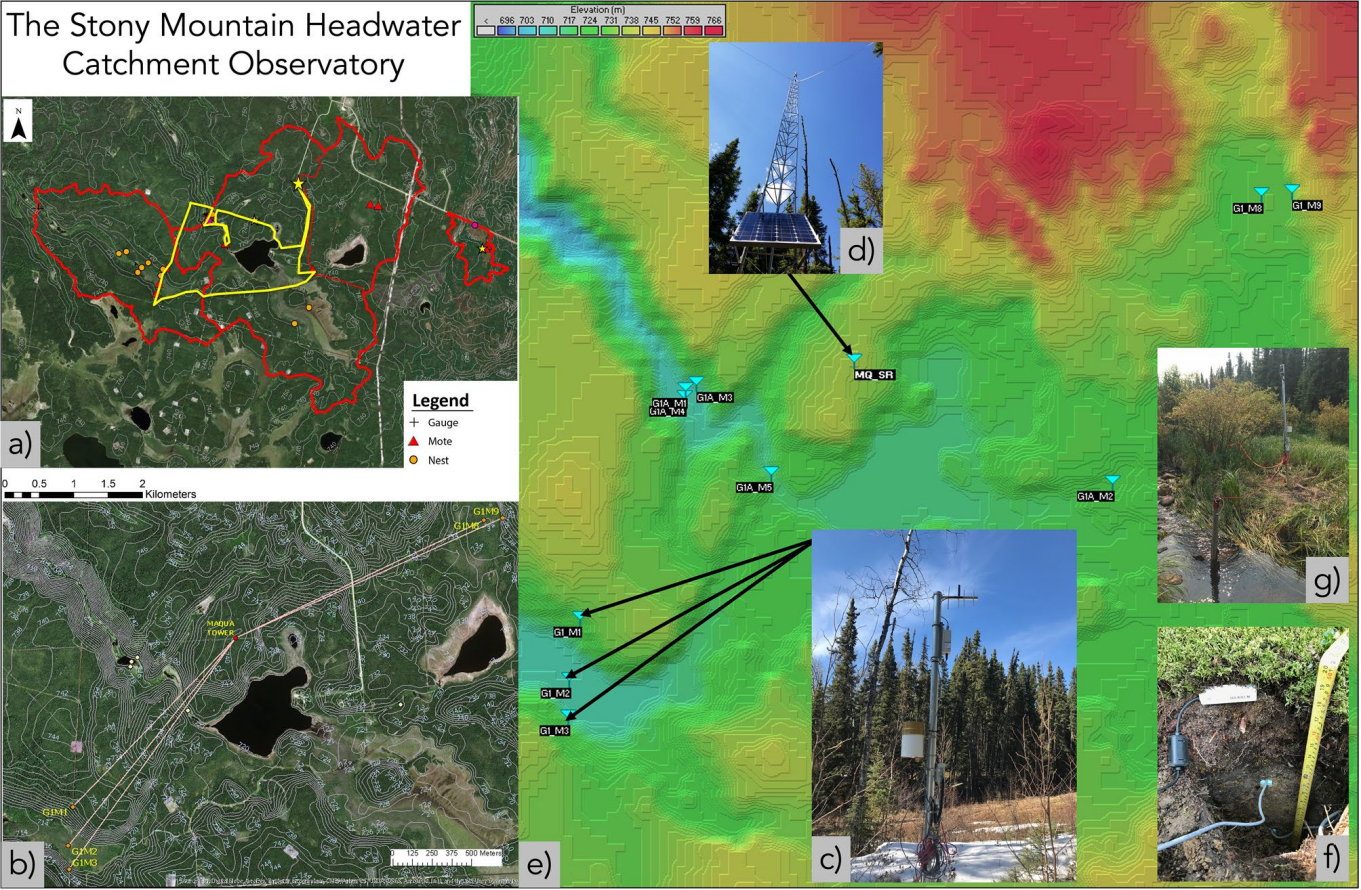
LPWAN deployment at SMHCO (maps and imagery generated using ESRI ArcGIS Pro 2.2; www.esri.com). (**a**) Map with study catchments and measurement locations. Yellow line indicates walking route to access remote measurement locations (> 12 km return distance), which highlights the logistical difficulty of accessing some study sites. (**b**) Direct communication lines between several motes, including topographic contours. (**c**) Mote configuration in the field (~ 2.5 m height), with arrows indicating locations of several motes within the SMHCO study area. (**d**) The central Maqua Lake gateway tower (~ 10 m height). (**e**) Digital elevation model of the study area including location of distributed motes. (**g**) mote at catchment outlet for streamflow (stage) measurements. f) SDI-12 soil moisture sensors installed at a mote.

The spatially distributed LPWAN infrastructure deployed at the SMHCO includes 17 sensor clusters or measurement locations and three central towers spanning the five study catchments (Figs. 1 and 2; Table 1). Each measurement location includes one Riot mote (slave, on 2.5 m mounting post) comprising a standalone cluster of up to 10 SDI-12 compatible sensors and a radio communication device and antenna, all powered by an internal 36 Ah power source (two 18 Ah 3.6 V primary batteries) housed within the mote. A central gateway (master, on 10 m radio tower; Fig. 2) requests and receives measurements from the SDI-12 sensors via data transmission packages from the motes. The gateway is powered and controlled by a traditional datalogger (Campbell Scientific CS-310), and data transmissions received at the gateway from the motes are subsequently stored on the datalogger, which was also configured with a 4G cellular modem for telemetry capabilities.

**Table 1:** SMHCO LPWAN configurations and communication distances for each mote. Note that the Maqua Lake mid and long range gateways are located on the same central tower.

Network Name (Gateway antenna type; gain)	Mote ID	Mote antenna type; gain (dBd)	Distance from gateway (m)
Pauciflora Basin: short-range motes (omnidirectional; 0.56 dBd)	G1M5	Omnidirectional (whip); 0.56	49
	G1M6	Omnidirectional (whip); 0.56	22
	G1M7	Omnidirectional (whip); 0.56	51
Meadow Creek: mid-range motes (omnidirectional; 6 dBd)	G2M1	Omnidirectional (whip); 0.56	1093
	G2M2	Omnidirectional (whip); 0.56	596
	G2M3	Omnidirectional (whip); 0.56	640
	G2M4	Omnidirectional (whip); 0.56	624
Maqua Lake: mid-range motes (omnidirectional; 3 dBd)	G1AM1	Omnidirectional (whip); 0.56	679
	G1AM2	Omnidirectional (whip); 0.56	1129
	G1AM3	Omnidirectional (whip); 0.56	635
	G1AM4	Omnidirectional (whip); 0.56	690
	G1AM5	Omnidirectional (whip); 0.56	552
Maqua Lake: long-range motes (omnidirectional; 6 dBd)	G1M1	Directional (Yagi); 6	1490
	G1M2	Directional (Yagi); 6	1694
	G1M3	Directional (Yagi); 6	1809
	G1M8	Directional (Yagi); 6	1743
	G1M9	Directional (Yagi); 6	1858

The current deployment is based on RT-designed LoRa wireless SDI-12 bridge units. The SMHCO implementation involves having the gateway intelligently estimate wireless and network introduced delay to the mote while simulating a sensor response to the connected datalogger. The gateway masquerades as an individual sensor to the datalogger (regardless of where the sensor is in the network topology) by responding quickly to the directly connected datalogger to meet the SDI-12 response time requirements, then calculates estimated sensor response (with network delay) and communicates to the datalogger an estimated data response time from the sensor. The datalogger will then wait the appropriate time for a delayed response. Retry mechanisms in the mote—sensor interface, and the gateway—mote interface are built in to provide maximum resiliency. The protocol configuration allows for multiple delay parameters (power on delay for sensors, etc.). In other words, the gateway acts as an SDI-12 wireless bridge and responds immediately to measurement commands on behalf of configured remote sensors. It uses a learning algorithm to dynamically adjust the SDI-12 measurement (i.e., M!) command responses to increase the sensor measurement times sufficient to include necessary delays for wireless transmission to/from the mote and time for the remote motes to power up the sensor, perform the requested measurement and return the measurement values to the gateway. The gateway issues the SDI-12 service request to the logger once all measurement values have been received from the remote sensor and are ready to be served to the logger.

The nature of the specific command and parameters that can be set are available by request from RT through www.riotwireless.com or the corresponding author.

**Figure 3:**
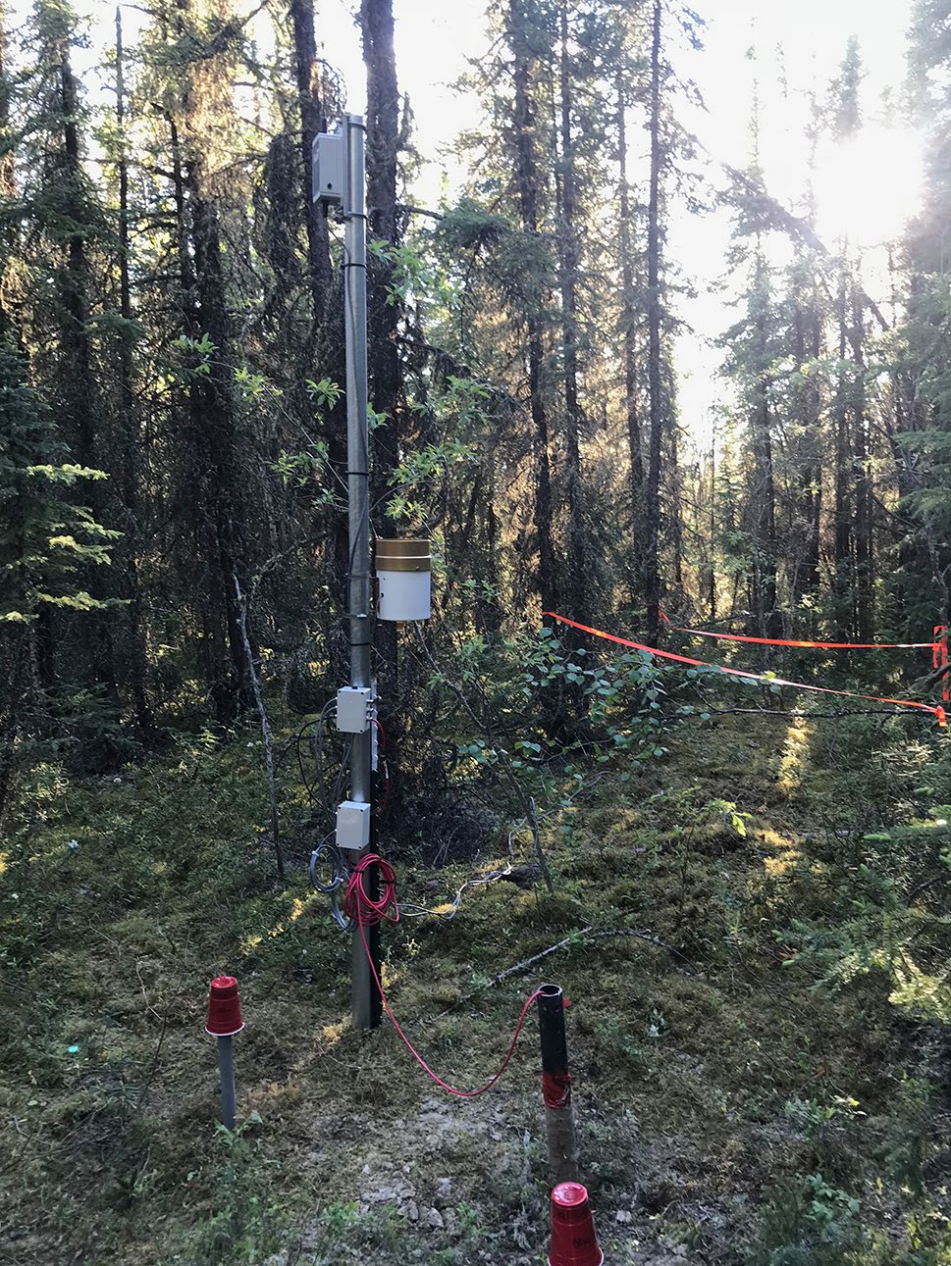
Typical mote configuration at SMHCO.

Communication distance between mote and gateway in the current deployment ranged from 22 to 1858 m (Table 1). In mote-gateway configurations where communication distance was approximately 1 km or less, 0.56 dBd omnidirectional whip antennae were used on the motes, and where communication distances were much greater (i.e., ~ 1.5–2.0 km), 6 dBd directional YAGI antennae were used, and aimed directly at the gateway (Table 1). Specific sensors deployed at each mote in this study included one conductivity/temperature/depth sensor (Meter Hydros-21) for measurement of water level (either groundwater elevation or stream stage), water temperature and electrical conductivity; three soil moisture probes (1 × Meter Teros-12 and 2 × GroPoint Lite) for volumetric water content, soil temperature and soil electrical conductivity (Teros-12 only); and a tipping bucket-type rain gauge (TR-525I) paired with a SDI-12 rain gauge interface (Tekbox TBSRGC1/2-SDI12; required to convert the “pulse” signal output from the rain gauge to a SDI-12 signal compatible with the motes). A typical mote and instrumentation setup is shown in Fig. 3.

## Methods

### Landscape terrain analyses and radio modelling

Modelling of the radio communication for each network was performed to analyse the effects of terrain and land cover on signal propagation between gateways and motes. A freeware software called Radio Mobile^19^, which employs the Longley-Rice radio propagation model^20^, was selected. The Longley-Rice radio propagation model is used to evaluate and predict radio transmission losses over irregular terrain relative to free-space transmission loss^20,21^. An elevation map with 30 × 30 m resolution was imported to Radio Mobile, which also accounts for land cover classification. The land cover dataset input for Radio Mobile was created based on merging of two separate datasets; a dataset containing forest types and their distribution (Alberta Wall-to-Wall land cover map, c.2010) and a more detailed LiDAR-derived dataset containing shape-files of bogs, fens, and marshes (Derived Ecosite Phase, 2017). The output was then reclassified to match the existing pre-set land cover types within Radio Mobile. Radio Mobile estimated the effects of hills and forest stand characteristics on communication and identified areas within the landscape where the weakest signal in communication was found (signal with strength of -130 dBm and lower would not be received). A number of different radio parameters necessary for radio propagation modelling were also configured in Radio Mobile; including transmitter power (Watt/dBm), receiver threshold (uV/dBm), line/wiring loss (dB), antenna type, antenna gain (dBi/dBd), and antenna height (m). Through integration and application of the Longley-Rice radio propagation model, radio signal strength was estimated, effectively incorporating the effect of the terrain elevation and landcover classification, including the height and density of vegetation, and the specific antennae configurations employed in each mote-gateway configuration.

### Weather conditions

Meteorological data were collected at a weather station within one of the SMHCO catchments that was operated independent of the LPWAN configuration (to provide weather variable data regardless of the LPWAN performance). Air temperature, relative humidity, windspeed and direction were measured every 60 s and hourly averages were recorded, along with cumulative hourly rainfall totals, using a Campbell Scientific CR1000 datalogger. This weather station was located within a SMHCO catchment approximately 3.5 km from the main LPWAN gateway tower (within the “Maqua Lake” network; Fig. 1), and is deemed as representative of the conditions there. This station was used primarily for rainfall analyses, as for most locations air temperature was taken as the external panel temperature of the datalogger (Campbell Scientific CS-310) that was located on the gateway tower to provide more locally-representative values. Rainfall analyses was not completed for the gateway and mote system that was further (~ 30 km) from this station (the “Meadow Creek” network; Fig. 1) due to increased proximity from rainfall measurements.

### Ethical responsibilities

All authors have read, understood, and have complied as applicable with the statement on "Ethical responsibilities of Authors" as found in the Instructions for Authors.

## Results

### Weather effects

Effects of air temperature and rain on the signal quality were assessed by analysing data collected between June 2018 to April 2019. During this time, air temperature ranged from − 40 to 29 ºC. Motes that were “short-range” had communication distances between the gateway and mote of < 100 m. “Mid-range” motes had gateway-to-mote communication distances of ~ 500 m to ~ 1.1 km, while “long-range” motes had the greatest distance of 1.5 to 2.0 km (Table 1). Communication “success” was classified as the receipt of the expected hourly transmission of a data packet from a mote containing all the data variables expected. Communication “failure” was deemed to have occurred when either no data was received by the gateway from a mote in any given hourly interval when one was expected, or when the mote was able to successfully transmit a partial data packet that only contained mote diagnostic information (e.g., mote battery voltage) but not the data variables from the sensors at the mote.

**Figure 4:**
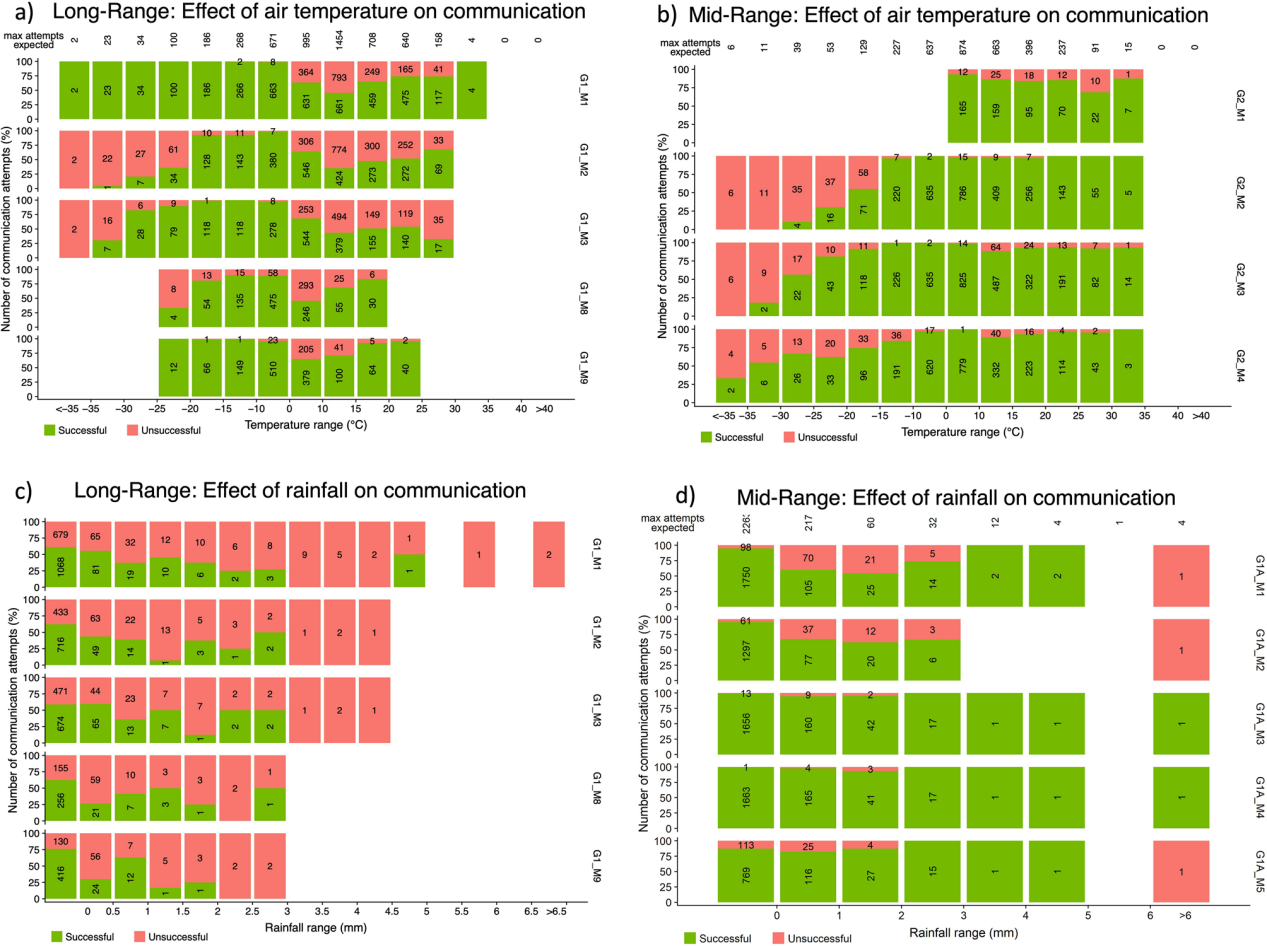
Effect of air temperature (**a**, **b**) and rainfall (**c**, **d**) on long and short-range communication success. The full bar represents all the data communication attempts (i.e., 100%; y-axis), with the green and red proportion of each bar representing successful and unsuccessful communication attempts, respectively. The numbers represent the actual count of communication attempts. Labels on the secondary y-axis identify the specific mote ID (see Tables 1 and 2 for mote details).

Temperature had the least impact on short-range mote communication successes, which rarely failed under both cold and warm air temperature conditions (communication success rate > 99%, not shown). Similarly, mid-range motes had very few instances of communication failure with warmer air temperatures. However, unlike the short-range motes, as air temperature dropped to below – 15 ºC, communication failure in the mid-range motes became apparent, with an increasing proportion of failed communication attempts as the temperature continued to drop to ~ − 35ºC (Fig. 4). For example, when air temperature ranged between – 10 and + 10 ºC, communication success among mid-range motes was over 98%, with only 63 out of a total of 4508 communication attempts unsuccessful. However, when the air temperature was between − 15 and – 20 ºC the communication success rate dropped to 74%. As the air temperature was further reduced, the success rates also declined, with average success rates reduced to 34% when air temperatures were less than – 20 ºC (Fig. 4). The long-range motes showed a similar trend as the mid-range motes, with reduced communication successes under the coldest air temperature conditions (Fig. 4). Owing to the less frequent nature of very cold air temperatures, the effective sample size of the number of attempted communication occurrences to evaluate under the coldest conditions is much less than the number of communication attempts to evaluate under more moderate air temperatures.

In contrast to the cold-temperature communication success observed, both the short and mid-range motes performed well under warm and hot air temperature conditions (i.e., air temperature 0–35 ºC). However, the long-range motes again displayed reduced communication successes with increasing air temperature; a trend which became apparent as soon as the air temperature exceeds 0 ºC. The highest proportion of communication failure occurred in the 10–15ºC range, where communication success was reduced to 55% (Fig. 4).

Rainfall was also observed to have an influence on the communication successes of the LPWAN systems, again showing a reduced but variable impact on the short and mid-range mote communication success rate, and a more apparent influence on the long-range mote data transmission success rate (Fig. 4). For example, when rainfall intensity was between 0 and 2 mm/h, mid-range mote communication success ranged from 59 to 97% success rates, with an average of 81% successful data transmission. For the long-range motes, data communication success was more of a challenge at all rainfall intensities, with near-zero communication success at rainfall intensities exceeding 3 mm/h (although, as with the extreme temperatures, the sample size of system performance under higher intensity rainfall intensity is greatly reduced due to the less-frequent occurrence of these conditions), and an average data communication success rate of 43% when rainfall intensity was between 0 and 2 mm/h (Fig. 4).

### Landscape terrain and topography

**Figure 5:**
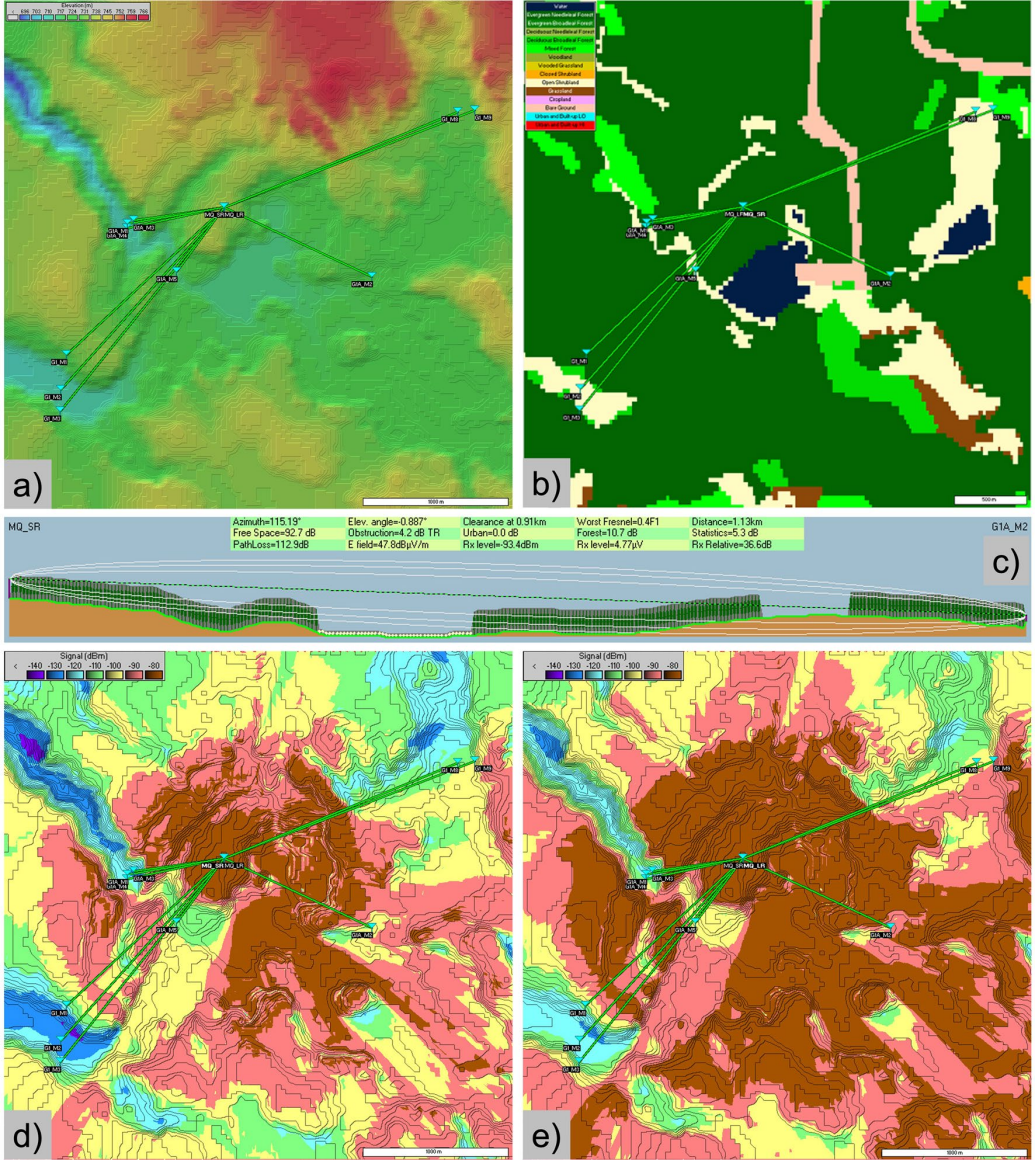
(**a**) Digital elevation model of the terrain surrounding the central Maqua Lake gateway tower (map generated using ESRI ArcGIS Pro 2.2; www.esri.com). (**b**) Land cover classifications used in Radio Mobile modelling. The land cover dataset input for Radio Mobile was created based on merging of two separate datasets; a dataset containing forest types and their distribution (Alberta Wall-to-Wall land cover map, c.2010) and a more detailed LiDAR-derived dataset containing shape-files of bogs, fens, and marshes (Derived Ecosite Phase, 2017). (**c**) Example of elevation and land cover profile and radio propagation modelling parameters generated by Radio Mobile, including the estimated ellipsoidal Fresnel zone. (**d**) Modelled received signal strength indicator (RSSI) for mid-range (**d**) and long-range (**e**) gateway antennae (Radio Mobile, RmWeb 2.1.2.0; http://www.ve2dbe.com/english1.html, 2020).

Each communication pathway from mote to gateway represents a unique set of topographic and vegetation conditions, since although the gateway is centrally located, the motes are spatially distributed throughout the study catchments (Fig. 5). Accordingly, there are 17 unique communication pathways that can be evaluated to understand the controls on communication success regarding topography. For consistency and comparability, the focus here will be on the “Maqua Lake” LPWAN Network, evaluating the performance of both the mid-range (552–1129 m communication pathway) and the long-range (1490–1858 m) motes (Figs. 1 and 5; Table 1).

Physical obstructions such as hillslopes were identified along many of the mote-to-gateway communication pathways where surface elevation rose to obstruct the direct line of sight. These obstructions resulted in strong signal loss and relative signal strength (Rx relative) that varied between 48.2 dB (G1M9; 1.9 km, unobstructed; Table 2) and 18.2 dB (G1M2; 1.7 km, topographic obstruction present at 1.47 km; Table 2) and was more important than physical distance along communication pathways for signal strength and degradation. For reference, the short-range mote Rx relative was as high as 91 dB at a mote-to-gateway communication pathway of 22 m (G1M6; not shown). Obstructions caused by the forest canopy also resulted in loss of signal over forested areas; however, this was less than obstructions caused by terrain when a terrain obstruction was present (indicated by “obst” rather than “clear” in Table 2). The magnitude of the signal loss that was attributed to a physical terrain obstruction (“Obstruction (loss)”, Table 2) was, with only one exception, greater than the signal loss that is attributable to the signal loss within the forest canopy under obstructed conditions (“Forest (loss)”, Table 2). For example, the mote-to-gateway radio signal strength for mote G1M3 was reduced by 28.8 dB largely due to the presence of an obstruction (hillslope) at 1.37 km, while the signal strength was only reduced by 11.3 dB due to the signal loss within the forest canopy along the communication pathway (Table 2).

**Table 2:** Network configurations for the Maqua Lake mid and long-range motes, including radio parameters and signal model values for each mote-to-gateway communication pathway (i.e., radio link).

			MID-RANGE					LONG-RANGE				
Mote ID			G1AM1	G1AM2	G1AM3	G1AM4	G1AM5	G1M1	G1M2	G1M3	G1M8	G1M9
Communication Pathway Distance (m)			679	1,129	635	690	552	1,490	1,694	1,809	1,743	1,858
Parameter	Explanation	Units										
Azimuth	Angle of the radio link from the transmitter to the receiver	degrees	260.3	115.2	261.9	257.9	216.5	227.0	221.8	218.8	67.64	68.79
Free Space	Loss of signal in free space	dB	88.2	88.2	87.6	88.4	86.4	95.1	96.2	96.8	96.4	97.0
Path Loss	Combined loss of signal between transmitter and receiver	dB	122.6	112.9	116.3	127.0	129.3	124.7	147.0	142.1	130.0	117.1
Elev. Angle	Angle of radio link with respect to earth (down tilt is negative)	degrees	1.772	1.772	1.540	1.978	2.405	0.376	0.866	0.798	0.536	0.227
Obstruction (loss)	Loss of signal due to obstructions (terrain, hills)	dB	13.6	4.2	7.4	17.3	19.9	7.1	34.2	28.8	18.2	6.6
E field	Electric field estimated at the receiver antenna	dB (uV/m)	38.2	47.8	44.5	33.8	31.3	38.9	16.5	21.4	33.6	46.5
Obstruction/Clearance (distance)	Distance between transmitter and any obstruction (obst) or lowest clearance (clear)	km	0.51 (obst)	0.91 (clear)	0.52 (clear)	0.51 (obst)	0.32 (obst)	1.34 (clear)	1.47 (obst)	1.37 (obst)	0.90 (obst)	0.90 (clear)
Rx level	Value expected at the receiver	dBm	101.5	93.4	95.6	105.9	109.2	89.4	111.8	106.9	94.7	81.8
Worst Fresnel	Distance to ground expressed as a factor of F1 at the obstruction/worst clearance	F1	0.2	0.4	0.2	0.3	0.5	0.2	1.3	1.0	0.4	0.2
Forest (loss)	loss of signal over forested areas	dB	15.4	10.7	15.9	16.0	17.6	17.2	11.3	11.3	10.0	8.2
Distance	distance between the transmitter and receiver	km	0.68	1.13	0.63	0.69	0.55	1.49	1.69	1.80	1.74	1.85
Statistics	Standard deviation of signal over the distance from the transmitter to receiver	dB	5.4	5.3	5.4	5.4	5.4	5.3	5.3	5.3	5.3	5.3
Rx Relative	Relative signal strength with respect to the Rx sensitivity	dB	28.5	36.6	34.4	24.1	20.8	40.6	18.2	23.1	35.3	48.2

### Longley-Rice radio propagation model

The output of the Longley-Rice radio propagation modelling analyses, completed using Radio Mobile software, provides spatially distributed maps of the predicted strength of the radio signal, as represented by the metric “Received Signal Strength Indicator” (RSSI; Fig. 5). RSSI is a relative index to measure the quality of the received signal, where higher values indicate better signal strength. The modelled RSSI throughout the LPWAN network configurations across the different study sites ranged from approximately − 80 to − 140 dBm. Functionally, a RSSI value of − 80 to − 100 dBm is strong, − 100 to − 120 dBm is acceptable, − 120 to − 130 dBm is weak, and < − 130 dBm is very weak and unlikely to result in successful communication between transmitter and receiver. The highest, and therefore strongest, radio signal strengths were predicted to be in the regions nearest to the location of the gateway, with spatially degrading modelled RSSI with increasing distance from the gateway. These patterns of RSSI signal strength largely followed patterns in surface topography, with lower RSSI predicted in topographic lows and valleys, and higher RSSI values predicted on topographic highs and hilltops (Fig. 5). The Longley-Rice modelling also demonstrates the different patterns and predicted RSSI under different radio parameter configurations. For example, the Maqua Lake mid and long-range mote network employ 3 and 6 dBd omnidirectional antennae at the gateway, respectively. Both gateways are located on the same 10 m tower; however, they display much different patterns of modelled RSSI across the same topographical terrain owing to their different power priorities and radio signal shapes. Although the predicted RSSI appears better in the long range configuration, the shape of the signal from the antenna is more narrow and less suited for communication success in nearby topographic lows. Additionally, this modelling also considers the antenna configuration of the transmitters (motes). The directional YAGI antennae used for the long-range motes at the Maqua Lake network resulted in a boost of approximately 10 dBm for the RSSI (Fig. 5). Fortunately, the actual RSSI between a subset of mote and gateway radio linkage pathways measured directly in the field was, in all but one case, higher than the modelled RSSI (Table 3).

**Table 3:** Measured and predicted received signal strength indicator (RSSI) values for each mote-gateway radio linkage. Measured values represent the average of hourly RSSI measurements from the field between each mote and gateway. Predicted RSSI values represent the modelled RSSI value between the mote and gateway based on the Longley-Rice modelling results. Note RSSI was not measured at all mote-gateway linkages in the field due to programming issues.

Mote ID	Transmission Distance (m)	Predicted RSSI from Longley-Rice Model (dBm)	Measured RSSI between gateway and mote (dBm)
G1A M3	635	− 125	− 112
G1A M4	690	− 115	− 106
G1A M5	552	− 110	− 112
G1 M1	1490	− 125	− 109
G1 M2	1694	− 130	− 112
G1 M3	1809	− 115	− 111

## Discussion

High-intensity rainfall events caused near-total loss of data from long-range motes, while short and mid-range motes were largely unaffected by rainfall at all intensities encountered. Signal attenuation caused by reflection and refraction processes driven by rainwater droplets can increase linearly with rainfall rate^22^. Rainfall rates of less than 2–3 mm/h have been found to have a negligible effect on radio communication performance^23^. Although in the current deployment, performance and data communication success rates dropped under moderate rainfall intensities for long-range motes (i.e., 43% success rate for rainfall intensity < 2 mm/h; Fig. 4), the frequency of the occurrence of higher intensity rainfall events is relatively low, so the overall impact of data communication failures at higher intensity rainfall events is minimal.

With respect to air temperature, short and mid-range motes performed well at temperatures above – 15 ºC, with communication failures becoming more common in mid-range motes below this threshold (e.g., communication success rates reduced to 34% when air temperatures were below – 20 ºC; Fig. 4). The communication success rates in the long-range motes demonstrated a bimodal distribution, with reduced communication successes under cold (< − 15 ºC) and warm (> 0 ºC) air temperatures (Fig. 4). Considering the infrequent occurrence of high-intensity rainfall events, and more reduced communication successes under air temperatures more commonly encountered, we can conclude that temperature was a stronger control on the communication success of the LPWAN system than was rainfall.

Although the batteries used within the deployment and the testing are rated to − 60 °C, the voltage levels required for reliable system operations sporadically drop below sustainable operating voltage due to the chemical changes within the batteries. Due to the battery mass, at lower temperatures, the chemistry of the cells does not have time to recover to the sufficient voltages after being cooled, resulting in a sustained period of sporadic operation at temperatures < − 15 ºC. Although the batteries have sufficient capacity with respect to amp hours (18 Ah each, total 36 Ah per mote); the voltage degradation at cold temperature degrades sufficiently to stop the system. Replacing the batteries alone does not solve the issue; however, a combination of higher grade, low temperature battery and energy harvesting/recharging capability (extreme cold 6.4 Ah internal battery pack with charging components and protection circuitry) have since been deployed in the field. While the replacement power system has less static capacity it has better voltage capacity. The lost overall capacity was replaced with energy harvesting capability, with better cold-weather performance observed in the field (data not shown).

However, there are other variables that are likely to have influenced the warm air temperature performance of the motes. For example, the time of year when air temperature exceeds 0 ºC and reaches consistently into the 20 ºC range is also when deciduous trees have leaves on their branches and, as such, the effective tree canopy is much denser and more obstructed than during the winter months with cold temperatures and only conifer tree canopies and bare branches of deciduous trees to contend with. Additionally, atmospheric perturbations and distortions, or scintillations, become more common as air temperature increases and refraction becomes more apparent^24^. Accordingly, under warming temperature, these disturbances were likely to have had an increasing impact on distorting and disturbing the radio communication signal along its path from transmitter (mote) to receiver (gateway). This was only an issue at the long-range motes (i.e., communication pathways of 1.5 to 2 km), as the short and mid-range motes did not display the same pattern of reduced communication successes with warmer air temperatures (Fig. 4). Considering this lack of impact at the short and mid-range scale, it can be concluded that the combined impact of forest stand density/canopy phenology (i.e., the presence or absence of deciduous leaves) and atmospheric disturbances (i.e., refraction and/or scintillations) are inconsequential for communication pathways of less than 1 km.

The key control on the strength of the signal and associated data transmission success was the presence or absence of physical obstructions caused by topographic variability along the communication pathway. The best data transmission was observed for motes that had no topographic obstructions along their mote-to-gateway radio link pathway (e.g., G1M1 and G1M9 in Table 2). However, when the local terrain rose to elevations that intersected the direct line of sight, such as when a hillslope is present within the communication pathway, the obstructions resulted in strong loss of signal. These challenging topographic conditions were compounded by the scientific need to instrument ecosystems (wetlands, streams) that typically occupy local topographic lows (Fig. 2). However, the presence of the Fresnel zone (i.e., the ellipsoidal region of space between the transmitter and receiver caused by radio waves following slightly different pathways;^25^; Fig. 5), helped to offset this signal loss and allow for continued successful data transmission, even along communication pathways where direct line of sight is obstructed (e.g., G1M2, Table 2). Consequently, consistently higher actual RSSI values were observed in the field as compared to those predicted using the Longley-Rice modelling. Radio communication waves undergo complex and diverse propagation mechanisms (e.g., diffraction, scattering, reflection, refraction) between the transmitter and receiver, with the received signal strength a cumulative sum of the propagations^26^. Although this radio propagation typically results in loss of signal strength, the functional cumulative effect of signal propagation observed in the field appears to be less than that predicted by the Longley-Rice model, which resulted in much more favourable (i.e., higher) RSSI values than expected, and increased successful data transmission rates above what would be expected based on the radio modelling alone.

The modelled reduction in RSSI demonstrates the predicted spatial patterns where relatively strong (high RSSI) and very weak (low RSSI) radio signal strengths can be expected (Fig. 5). The lower RSSI values (e.g., − 120 to − 130 dBm in Fig. 5) were driven primarily by topographic constraints and signal degradation attributed to surface terrain variability, owing to the greater impact of physical obstructions caused by terrain on signal degradation (when present) than the signal reductions caused by interference with the forest canopy (Table 2).

Based on this study, a few recommendations for future application of similar LPWAN deployments can be made. Firstly, it is highly advisable to take the time and model the potential study sites for suitability for LPWAN deployment and specific site selection before going to the field. All the information needed to build the Longley-Rice radio propagation model can be determined without the need for an actual investigation in the field. Digital elevation models and landscape classifications are typically available, and radio parameter selections can be made and optimized based on the outcome of the modelling exercise. Then, specific study site selection can be guided based on the overall landscape positioning and anticipated radio communication signal strengths in the general areas of interest. Fortunately, our results demonstrated higher RSSI in real-world application as compared to the modelled scenarios (Table 3); however, this exercise will surely save time and effort while instrumenting selected sites in the field and will help to ensure optimal radio communication thereafter. Additionally, a favourable technology advancement would be to integrate on-board data storage capability on the motes or transmitters. In the current deployment, if the data packet failed to send over three subsequent attempts, the data were lost. However, on-board storage of data would provide some resiliency against data loss during challenging communication conditions (e.g., high or low air temperatures, rainfall events; Fig. 4). Additionally, LPWAN networks can offer “repeater” motes to integrate more radio communication resiliency into LPWAN deployments. These repeater motes essentially receive and re-transmit data packets and relay them on from the original transmitter or mote to the end receiver or gateway. This provides a realistic option to help overcome some of the challenges presented by terrain features such as hills, which are often unavoidable in environmental applications.

Although there are many implementations of LPWAN devices connecting to SDI-12 sensors and transmitting that data to gateway infrastructures, the novelty of this implementation is the fact that a standard SDI-12 datalogger can be set up to seamlessly access remote sensor data over the LPWAN. The current deployment allows the existing environmental monitoring industry SDI-12 dataloggers to extend their reach, as it imitates a wired connection to the sensor, thereby allowing the industry standard dataloggers to be used. LPWAN systems also offer a cost–benefit, as they can be deployed at a much lower cost than traditional data logger and instrumentation configurations, and each mote and associated sensor cluster has an internal power source. Additionally, with the majority of the data transmission occurring over the local radio network, telemetry is only required at the central gateway station, to enable near real-time data collection at numerous spatially-distributed measurement locations.

## Conclusions

The LPWAN system performance evaluated in this study was negatively impacted by very low (less than – 15 ºC) and warm (> 0 ºC) air temperatures and rainfall over longer distances that exceeded ~ 1.4 km. However, shorter-range communication distances (< 1.2 km) consistently performed well, with optimum system performance observed in the 0–700 m range. Temperature was a stronger control on the communication success of the LPWAN system than was rainfall; however seasonal influences including canopy density characteristics driven by deciduous tree leaf cycles were also likely a factor in decreased communication successes over distances exceeding ~ 1 km during times with warmer air temperatures. Data transmission was strong across communication distances of less than ~ 1 km, with inconsequential influences of forest stand density, canopy phenology and atmospheric disturbances. Data transmission interference caused by topographic features obstructing communication line of sight was the predominant cause of data loss. The performance of the beta version of this LPWAN technology in challenging terrain and vegetation cover exceeded the expected capability of older Wireless Sensor Network technologies that do not employ LPWAN technology. This newer LPWAN technology can provide access to near real-time data from difficult-to-access areas in challenging terrain, over distances nearing 2 km. Accordingly, LPWAN technology can facilitate a process-based understanding of remote hydrological setting and represents a substantial advancement to environmental measurement and monitoring applications.

## Data Availability

The datasets generated during and/or analysed during the current study are available from the corresponding author on reasonable request.

## References

[1] Malek S (2017). Real-time Alpine measurement system using wireless sensor networks. Sensors.

[2] Kerkez B, Glaser SD, Bales RC, Meadows MW (2012). Design and performance of a wireless sensor network for catchment-scale snow and soil moisture measurements. Water Resour. Res..

[3] Bogena HR (2010). Potential of wireless sensor networks for measuring soil water content variability. Vadose Zone Journal.

[4] Burt TP, McDonnell JJ (2015). Whither field hydrology? The need for discovery science and outrageous hydrological hypotheses. Water Resour. Res..

[5] Peel MC, Blöschl G (2011). Hydrological modelling in a changing world. Progress Phys. Geogr..

[6] Devia GK, Ganasri BP, Dwarakish GS (2015). A review on hydrological models. Aquatic Procedia.

[7] Beven KJ (2000). Uniqueness of place and process representations in hydrological modelling. Hydrol. Earth Syst. Sci..

[8] Thirel G, Andréassian V, Perrin C (2015). On the need to test hydrological models under changing conditions.. Hydrol. Sci. J..

[9] Zia H, Harris NR, Merrett GV, Rivers M (2019). A low-complexity machine learning nitrate loss predictive model—Towards proactive farm management in a networked catchment. IEEE Access.

[10] Zia H, Harris N, Merrett G, Rivers M (2015). Predicting discharge using a low complexity machine learning model. Comput. Electron. Agric..

[11] Eum H-I, Dibike Y, Prowse T (2017). Climate-induced alteration of hydrologic indicators in the Athabasca River Basin, Alberta, Canada. J. Hydrol..

[12] Shrestha NK, Du X, Wang J (2017). Assessing climate change impacts on fresh water resources of the Athabasca River Basin, Canada. Sci. Total Environ..

[13] Shope CL (2014). Using the SWAT model to improve process descriptions and define hydrologic partitioning in South Korea. Hydrol. Earth Syst. Sci..

[14] Hrachowitz M (2013). A decade of Predictions in Ungauged Basins (PUB)—A review. Hydrol. Sci. J..

[15] Sivapalan M (2003). IAHS decade on predictions in ungauged basins (PUB), 2003–2012: Shaping an exciting future for the hydrological sciences. Hydrol. Sci. J..

[16] Sieber, A. *et al.* in *2008 International Workshop on Intelligent Solutions in Embedded Systems.* 1–14 (IEEE).

[17] Sieber A, Markert J, Woegerer C, Cocco M, Wagner MF (2010). Advances in Wireless Sensors and Sensor Networks.

[18] Natural Regions, C. Natural regions and subregions of Alberta, Pub. no. *T/852. Government of Alberta* (2006).

[19] Radio Mobile, Roger Coudé v. RmWeb 2.1.2.0 (http://www.ve2dbe.com/english1.html, 2020).

[20] Longley AG (1968). Prediction of Tropospheric Radio Transmission Loss Over Irregular Terrain: A Computer Method-1968.

[21] Shepherd NH (1988). Coverage prediction for mobile radio systems operating in the 800/900 MHz frequency range. IEEE Trans. Veh. Technol..

[22] Nadeem, F., Chessa, S., Leitgeb, E. & Zaman, S. The effects of weather on the life time of wireless sensor networks using FSO/RF communication. *Radioengineering* **19** (2010).

[23] Boano, C. A., Brown, J., He, Z., Roedig, U. & Voigt, T. *Sensor Applications, Experimentation, and Logistics: First International Conference, SENSAPPEAL 2009, Athens, Greece, September 25, 2009, Revised Selected Papers 1.* 159–176 (Springer).

[24] Deaconu C (2018). Measurements and modeling of near-surface radio propagation in glacial ice and implications for neutrino experiments. Phys. Rev. D.

[25] Osterman A, Ritosa P (2019). Radio propagation calculation: A technique using 3D Fresnel zones for decimeter radio waves on LiDAR data. IEEE Antennas Propag. Mag..

[26] Neskovic A, Neskovic N, Paunovic G (2000). Modern approaches in modeling of mobile radio systems propagation environment. IEEE Commun. Surv. Tutor..

